# Antimicrobial susceptibility profiles of *Staphylococcus aureus* in cattle and humans in farming communities of Isingiro and Kamuli districts, Uganda

**DOI:** 10.1038/s41598-024-52035-1

**Published:** 2024-01-22

**Authors:** Joseph M. Kungu, Sarah S. Tegule, Ilyas A. Awke, Josephine Namayanja, Edity Namyalo, Joseph Oposhia, William Olum, Luke Nyakarahuka, Clovice Kankya, Dieudonné Dahourou, Agricola Odoi

**Affiliations:** 1 College of Veterinary Medicine Animal Resources and Biosecurity, Makerere University, Kampala, Uganda; 2 Ministry of Agriculture Animal Industry and Fisheries, Entebbe, Uganda; 3 Ministry of Health, Kampala, Uganda; 4 University of Dedougou, Ouagadougou, Burkina Faso; 5 Department of Biomedical and Diagnostic Sciences, University of Tennessee, Knoxville, USA

**Keywords:** Microbiology, Risk factors

## Abstract

Bacterial resistance to antimicrobials is fast becoming a big challenge as resistance to multiple drugs is rising rapidly. The emergence of resistant *Staphylococcus aureus* worldwide is life-threatening in both humans and animals and yet little is known about the burden of antimicrobial resistance (AMR) in developing countries including Uganda. Therefore, the aims of this study were to determine the prevalence of antimicrobial resistant *S. aureus* among humans and animals as well as assess the perceptions and practices of farmers in Kamuli and Isingiro districts in Uganda regarding AMR of *S. aureus*. A cross-sectional study was conducted between July and September 2020 in 147 randomly selected cattle-keeping households in Isingiro and Kamuli districts. A structured questionnaire uploaded in the Kobo-collect online data collection tool was used to assess farmers’ perceptions and practices pertaining to AMR in each of the selected households. Nasal swabs (n = 147) were collected from both cattle and humans (farmers). Bacterial isolation and confirmation was done using Gram-staining and biochemical tests. This was followed by antimicrobial susceptibility testing (AST) using the Kirby Bauer disc diffusion method. Only 14/147 (9.5%) cattle samples and 45/147(30.6%) human samples tested positive for *S. aureus*. All cattle *S. aureus* isolates were resistant to Nitroimidazoles while 92.9% were resistant to Penicillins. None of the isolates were resistant to Fluoroquinolones and Aminoglycosides. All the 14 isolates exhibited AMR to at least one of the assessed antibiotics and 92.9% (13/14) showed evidence of multidrug resistance (MDR). Likewise, *S. aureus* human isolates showed high levels of resistance to Nitroimidazoles (100%) and Penicillins (93.3%), with none of the isolates having resistance to Aminoglycosides, and only one exhibiting resistance to Fluoroquinolones (2.2%). All the 45 human isolates exhibited AMR to at least one antibiotic while 93% (42/45) had MDR. Most farmers had good perceptions of AMR, with a significantly higher proportion of respondents from Isingiro than Kamuli showing a better understanding of AMR. Antibiotic prophylaxis was reported to be the least practiced measure of diseases and parasites control (17.0%), with more farmers in Isingiro (33.3%) undertaking it than those in Kamuli (1.3%) (p < 0.001). Penicillins and Nitroimidazoles were reported to be the most used antibiotics among cattle and humans. This study provides evidence of occurrence of *S. aureus* resistance to antimicrobials commonly used in both humans and livestock in Isingiro and Kamuli districts. Farmers had good perceptions regarding AMR as well as good antimicrobial use practices which can form a basis for mitigation of AMR.

## Introduction

Antimicrobial resistance is increasingly becoming a big challenge to healthcare systems worldwide as the pace of development and spread of resistance genes far overwhelms the pace of development of new drugs^1^. Several studies have documented occurrence of AMR among commensal and pathogenic bacteria globally^2–7^. Studies conducted in African countries have revealed an increasing prevalence of antimicrobial resistance (AMR) among *staphylococcus aureus* isolates^7–9^. A study by the *Uganda National Academy of Sciences* revealed increasing trends of AMR among *S. aureus* isolates to the most commonly used antibiotics such as Penicillins, Cotrimoxazole, and Tetracyclines^10^. Of particular concern was presence of multi-drug resistant bacteria such as extended-spectrum beta-lactamase (ESBL) and methicillin-resistant *staphylococcus aureus* (MRSA)^10^.

In Uganda, treatment of bacterial infections in both humans and animals is largely empirical with limited use of antibiograms to guide therapy^2,8,11^. Moreover, the availability of over-the-counter antimicrobials in unlicensed drug shops, or open markets and the lack of enforcement of drug-use laws facilitates injudicious use of antimicrobials^12,13^. This abuse of antimicrobials in different settings potentially contributes to the rise in the burden of AMR and multidrug resistant microorganisms. Unfortunately, there is sparse data on the burden of AMR in both human and animal populations as well as the environment^2,12,14^ and yet this information is of critical importance to guide both policy and control programs. Therefore, the the study aimed at determining the prevalence of antimicrobial resistant *S. aureus* among humans and animals as well as assess the perceptions and practices of farmers in Kamuli and Isingiro districts in Uganda regarding AMR of *S. aureus*.

This information will be critical for guiding policy decisions aimed at curbing antibiotic abuse in both animal and human medicine and hence combatting the problem of AMR.

## Methods

### Site selection

**Figure 1:**
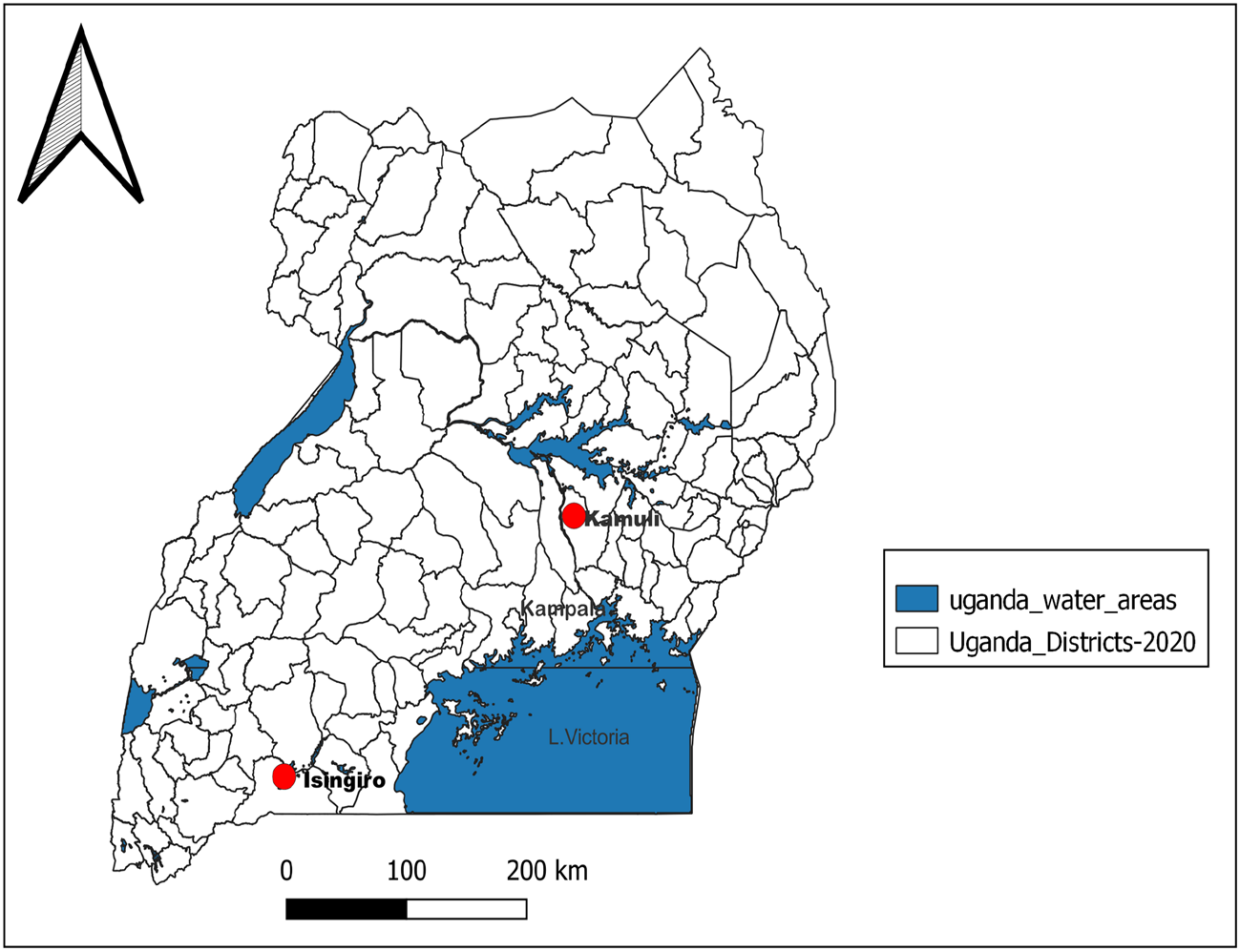
Map of Uganda showing study districts.

A cross-sectional study was conducted in Isingiro (majorly agro-pastoral farming system) and Kamuli (mixed farming system) districts between July and October 2020 (Fig. 1). Selection of the study sites (Districts and their Sub-Counties) was based on farming systems, population density of cattle, as well as burden of diseases in both humans and animals^15^. Isingiro district is in South Western Uganda, covering a land area of 2655.6 km^2^ with a population of 492,116 people, and with > 90% engaging in livestock farming^15^. Kamuli district is in the Eastern part of the country, covering a land area of 1557 km^2^ with a population of 500,800 people mostly involved in crop and livestock farming^15^. The two districts are among the key cattle keeping communities of Uganda and are known for endemicity of transboundary diseases such as Foot and Mouth diseases, Contagious Bovine Pleuro Pneumonea (CBPP) and mastitis. Farmers’ efforts to control these conditions result in uncontrolled use of antibiotics^16^.

### Sample size and sampling

The minimum required sample size of 97 households was estimated assuming a 50% prevalence of *Staphylococcus aureus,* allowable error of 10%, and an alpha of 5%. However, a total of 147 households (71 in Isingiro and 76 in Kamuli) were sampled to increase precision. A list of all cattle keeping households was generated by the Veterinary Officers in charge of the selected study sub-counties (Ndiizi and Rugaga in Isingiro as well as Kitayundwa and Balawoli in Kamuli district). The study households were then randomly selected using computer-generated random numbers. Apparently healthy cattle of age ≤ 6 months old and human participants at least 10 years of age who were directly involved in herding of cattle were selected following physical examination. One animal and one human participant fulfilling the eligibility criteria was randomly selected from selected household.

### Data collection

From each selected household, two nasal swabs (one human and another cattle) were collected using sterile cotton-tipped swabs, kept in Stuart media (manufacturer) at 4 °C and transported to Makerere University, College of Veterinary Medicine Animal Resources and Biosecurity for laboratory processing and analysis. A structured questionnaire in Kobo-Collect electronic software was used to capture demographic data of the selected cattle and human participants as well as the respondents’ perceptions and practices regarding antibiotic use and its potential contribution in the development of AMR.

The questionnaire was pretested in five cattle keeping households Bugulumbya Sub County, Kamuli district before use.

### Laboratory analysis of samples

#### Bacterial isolation and identification

The swab sticks were broken off letting the nasal swab to drop into a bijou bottle containing sterile buffered Peptone water (Conda, Spain). The bijou bottle was vortexed to release and homogenize the bacteria into the peptone water. Mannitol Salt Agar (MSA) (Conda, Spain) was used as the selective and differential medium for isolation of *S. aureus*. Mannitol Salt agar was prepared according to Manufacturer’s instructions and a sterility test was done by incubating the casted plates at 37 °C over-night. The homogenized samples were then inoculated unto the Mannitol salt agar by the streak method and incubated at 37 °C overnight. The American type culture collection cult- loops, ATCC 25923 (Oxoid, Hamphsire, England) were used as positive control. Suspect yellow colonies with yellow zones were selected and sub-cultured on salted nutrient agar (Conda, Spain) to get pure colonies of *S. aureus* that were then used for Gram staining and further identification by carrying out Catalase, Indole, Citrate and the Coagulase biochemical tests. Confirmed *S. aureus* cultures were preserved in in tryptone soya broth with 80% glycerol in cryo-vials in a Freezer for antimicrobial susceptibility testing.

#### Antimicrobial susceptibility testing

Antimicrobial susceptibility testing was done using the Kirby Bauer disc diffusion method whereby, pure colonies of a well-isolated organism were emulsified in 4 mls of 0.9% sterile saline with the turbidity equivalent to 0.5 McFarland standard. This was inoculated on Mueller Hinton Agar (MHA) by spreading the saline suspension of the colonies and left to dry for about 15 min. Discs of nine commonly used antimicrobials were used: ciprofloxacin (5 μg), gentamycin (10 μg), tetracycline (30 μg), amoxicillin (25 μg), vancomycin (30 μg), ampicillin (10 μg), erythromycin (15 μg), metronidazole (10 μg), and penicillin-g (10 μg) (Source: Oxoid, Basingstoke, UK). The incubation was carried out at 37 °C for 24 h. The zone of inhibition was measured based on the guidelines of the Clinical and Laboratory Standards Institute (CLSI). The interpretation of the measurement of the sensitive (S) Intermediate (I) and resistant(R) bacteria was based on the inhibition zone diameters interpreted in comparison with interpretative tables provided by the National Committee for Clinical Laboratory Standards (NCCLS, 2001).

### Statistical analyses

All statistical analyses were performed in R software (version 3.2.4). Descriptive statistics of the AMR patterns, farmers’ perceptions of AMR as well as the practices associated with AMR occurrence in the two districts were performed and compared using the Chi square tests.

### Ethical clearance

The study was approved by the Makerere University College of Health Sciences, Research and Ethics committee (MAKSHSREC-2020-12). Informed consent was obtained from all subjects and/or their legal guardian(s) before their participation in the study. All methods were carried out in accordance with relevant guidelines and regulations.

## Results

### Sociodemographic characteristics of farmers

Out of 147 farmers, 74.1% (n = 109) were male. There was, however, no significant (p = 0.375) differences in gender distribution of farmers in the two districts. Most (67.3%) of the respondents were 20–50 (mean = 39.8, SD = 14.8) years of age, with Isingiro having a significantly (p = 0.023) higher percentage in the category (74.6%) compared to Kamuli (60.5%).

The majority (85.7%) of the cattle in the study were female, with Isingiro having a significantly (p < 0.001) higher percentage (98.6%) compared to Kamuli (73.7%). The cattle were mostly cross breeds (76.2%), and Isingiro had a significantly (p = 0.022) higher percentage (84.5%) than Kamuli (31.6%). Most (77.4%) of the farmers in Isingiro had > 5 head of cattle while up to 82.9% of the farmers in Kamuli were smallholders, keeping 1–5 head of cattle (Table 1) with the percentages in the different categories varying significantly (p < 0.001).

**Table 1:** Socio-demographic characteristics of farmers and their cattle in Kamuli and Isingiro districts, Uganda.

Characteristic	Both districts, overall = 147, n(%)	Isingiro , overall = 71, n(%)	Kamuli, overall = 76, n(%)	p-Values
Gender of farmers				
Male	109 (74.1)	55 (77.5)	54 (71.5)	0.375
Female	38 (25.9)	16 (22.5)	22 (29.0)	
Age of farmers				
< 20 years	20 (13.6)	4 (5.6)	16 (21.1)	0.023
20–50 years	99 (67.3)	53 (74.6)	46 (60.5)	
> 50 years	28 (19.1)	14 (19.7)	14 (18.4)	
Sex of cattle				
Female	126 (85.7)	70 (98.6)	56 (73.7)	< 0.001
Male	21 (14.3)	1 (1.4)	20 (26.3)	
Cattle breed				
Cross breed	112 (76.2)	60 (84.5)	24 (31.6)	0.022
Local breed	35 (23.8)	11 (5.5)	52 (68.4)	
Age of cattle				
1–5 years	62 (42.2)	16 (22.5)	47 (61.8)	< 0.001
6–10 years	66 (44.9)	40 (56.3)	25 (32.9)	
> 10 years	19 (12.9)	15 (21.1)	4 (5.3)	
Herd size of cattle kept				
1–5	64 (43.5)	1 (1.4)	63 (82.9)	< 0.001
6–15	16 (10.9)	6 (8.4)	10 (13.2)	
> 15	67 (45.6)	64 (90.1)	3 (3.9)	

### Prevalence of S. aureus and its antimicrobial resistance

Of the 147 nasal swabs collected from cattle, only 14 (9.5%) tested positive for *S. aureus* and were assessed for AMR. All (100%) the *S. aureus* isolates assessed for antibiotic resistance were resistant to Nitroimidazoles while 92.9% were resistant to Penicillins. None of the isolates were resistant to Fluoroquinolones (0.0%) and Aminoglycosides (0.0%) (Table 2). All the 14 isolates exhibited antimicrobial resistance to at least one of the assessed antimicrobials and 92.9% (13/14) showed evidence of multidrug resistance (MDR).

**Table 2:** Antimicrobial resistance profiles of *S. aureus* isolated from cattle in Isingiro and Kamuli of Uganda.

Antimicrobial	Number of resistant isolates (overall = 14)	Percentage
Fluoroquinolones	0	0
Ciprofloxacin	0	0
Aminoglycosides	0	0
Gentamicin	0	0
Tetracyclines	7	50
Tetracycline	7	50
Penicillins	13	92.9
Amoxicillin	3	21.4
Ampicillin	13	92.9
Penicillin G	12	85.7
Glycopeptides	1	7.1
Vancomycin	1	7.1
Macrolides	3	21.5
Erythromycin	3	21.5
Nitroimidazoles	14	100
Metronidazole	14	100

For the human samples, 30.6% (45/147) of nasal swabs were positive for *S. aureus* and were assessed for antimicrobial susceptibility. *Staphylococcus. aureus* human isolates showed high levels of resistance to Nitroimidazoles and Penicillins (93.3%). However, none of the isolates were resistant to Aminoglycosides, and only 1 exhibited resistance to Fluoroquinolones (2.2%) (Table 3). All the 45 human isolates exhibited AMR to at least one antimicrobial while 93% (42/45) showed evidence of MDR.

**Table 3:** Antimicrobial susceptibility profiles of *S. aureus* isolated from humans in Isingiro and Kamuli of Uganda.

Antimicrobial classes	Number of resistant isolates (overall = 45 )	Percentage
Fluoroquinolone	1	2.2
Ciprofloxacin	1	2.2
Aminoglycosides	0	0.0
Gentamycin	0	0.0
Tetracyclines	23	51.1
Tetracycline	23	51.1
Penicillins	42	93.3
Amoxicillin	19	42.2
Ampicillin	41	91.1
Penicillin G	42	93.3
Glycopeptides	6	13.3
Vancomycin	6	13.3
Macrolides	10	22.2
Erythromycin	10	22.2
Nitroimidazoles	45	100
Metronidazole	45	100

### Perceptions and practices of farmers regarding AMR

#### Perceptions of farmers regarding AMR

Up to 69.7% (15.6% strongly agreed and 53.1% agreed) of the respondents believed that AMR occurs when bacteria in the body become resistant to antibiotics (Table 4). The percentage of respondents reporting this perception were significantly (p < 0.001) higher in Isingiro (15.5% strongly agreed, 73.2% agreed) than Kamuli (15.8% strongly agreed, 34.2% agreed). Slightly more than a half of the respondents (59.2%) either strongly agreed (11.6%), or agreed (47.6%) that infections are becoming increasingly unresponsive to antibiotic treatment. The percentages reporting this were also significantly (p < 0.001) higher in Isingiro (15.5% strongly agreed, 67.6% agreed) than Kamuli (7.9% strongly agreed, 28.9% agreed). A total of 62.5% of the farmers (15.6% strongly agreed and 46.9% agreed) believed that if bacteria are resistant to antibiotics, it can be very difficult or impossible to treat the infections they cause. The proportion of farmers with this perception were also significantly higher in Isingiro than Kamuli (Table 4). Suffice it to say that a higher proportion of the respondents in Isingiro tended to show understanding of AMR compared to those from Kamuli (Table 4).

**Table 4:** Perceptions regarding antimicrobial resistance among farmers in Isingiro and Kamuli districts of Uganda.

Statements on AMR	Both districts, overall = 147)	Isingiro (n = 71)	Kamuli (n = 76)	p-value
AMR occurs when bacteria in the body when bacteria in your body become resistant to antibiotics and they no longer work as well become resistant to antibiotics				
Strongly agree	23 (15.6)	11 (15.5)	12 (15.8)	< 0.001
Agree	78 (53.1)	52 (73.2)	26 (34.2)	
Neutral	43 (29.2)	7 (9.9)	36(47.4)	
Disagree	3 (2.0)	1 (1.4)	2 (2.6)	
Strongly disagree	0 (0)	0 (0)	0 (0)	
Infections are becoming increasingly unresponsive to treatment by antibiotics				
Strongly agree	17 (11.6)	11 (15.5)	6 (7.9)	< 0.001
Agree	70 (47.6)	48 (67.6)	22 (28.9)	
Neutral	56 (38.1)	9 (12.7)	47 (61.8)	
Disagree	2 (1.4)	2 (2.8)	0 (0)	
Strongly disagree	2 (1.4)	1 (1.4)	1 (1.3)	
If bacteria are resistant to antibiotics, it can be very difficult or impossible to treat the infections they cause				
Strongly agree	23 (15.6)	13 (18.3)	10 (13.2)	0.001
Agree	69 (46.9)	44 (62.0)	25 (32.9)	
Neutral	52 (35.5)	12 (16.9)	77 (52.6)	
Disagree	3 (2.0)	2 (2.8)	1 (1.3)	
Strongly disagree	0 (0)	0 (0)	0 (0)	
Bacteria that are resistant to antibiotics can be spread from humans to humans as well as animals to humans				
Strongly agree	39 (26.5)	10 (14.1)	29 (38.2)	< 0.001
Agree	64 (43.5)	47 (66.2)	17 (22.4)	
Neutral	38 (25.8)	9 (12.7)	29 (38.2)	
Disagree	6 (4.1)	5 (7.0)	1 (1.3)	
Strongly disagree	0 (0)	0 (0)	0 (0)	
Antibiotic-resistant infections could make medical procedures like surgery, organ transplant, and cancer treatment much more dangerous				
Strongly agree	12 (8.2)	8 (11.3)	4 (5.3)	< 0.001
Agree	69 (46.9)	49 (69.0)	20 (26.3)	
Neutral	56 (38.1)	10 (14.1)	46 (60.5)	
Disagree	10 (6.8)	4 (5.6)	6 (7.9)	
Strongly disagree	0 (0)	0 (0)	0 (0)	

#### Farmers’ practices associated with AMR

The farmers mostly consulted qualified professionals (66%) whenever their animals were sick. Significantly (p < 0.001) more farmers did this in Kamuli (98.7%) than in Isingiro (31%) (Table 5). Antibiotic prophylaxis was observed to be the least practiced measure of control of diseases and parasites (17.0%), with significantly (p < 0.001) more farmers in Isingiro (33.3%) undertaking it than those in Kamuli (1.3%).

**Table 5:** Farmers’ disease management and antimicrobial use practices in Isingiro and Kamuli districts of Uganda.

Practices	Both districts (n = 147)	Isingiro (n = 71)	Kamuli (n = 76)	p-values
When your animals are sick, what did you do?				
Consult someone with experience	17 (11.6)	17 (23.9)	0 (0)	** < 0.001**
Consult a qualified professional	97 (66)	22 (31.0)	75 (98.7)	** < 0.001**
Consult the Vet drug shop vendor	48 (32.7)	46 (64.8)	2 (2.6)	** < 0.001**
Use personal experience to treat	47 (32)	42 (59.2)	5 (6.6)	** < 0.001**
Use previous prescription	18 (12.2)	17 (23.9)	1 (1.3)	** < 0.001**
Use traditional or Herbal remedies	3 (2.0)	3 (4.2)	0 (0)	0.07
Which methods do you use to prevent disease in your cattle?				
Probiotic	0 (0)	0 (0.0)	0 (0.0)	**–**
Antibiotic Prophylaxis	25 (17.0)	24 (33.8)	1 (1.3)	** < 0.001**
Vaccination	107 (72.8)	66 (93.0)	41 (53.9)	** < 0.001**
Deworming	76 (51.7)	35 (49.3)	41 (53.9)	0.469
Spraying or dip	142 (96.6)	68 (95.8)	74 (97.4)	0.590
No treatment	0 (0)	0 (0.0)	0 (0.0)	**–**
What happens when a lactating cow undergoes antibiotic treatment?				
Keep milking it and consuming the milk	17 (11.6)	11 (15.5)	6 (7.9)	0.150
Only give milk to calves for the next few days	37 (25.2)	20 (28.2)	17 (22.4)	0.418
Do not consume the milk for > 7 days after treatment	80 (54.4)	51 (71.8)	29 (38.2)	** < 0.001**

Significant values are in [bold].

More than half (54%) of the farmers reported that they followed drug withdrawal periods by not consuming milk from lactating cows for > 7 days after treatment and this practice was significantly (p < 0.001) different in the two districts (71.8% in Isingiro, 38.2 in Kamuli) (Table 5).

### Antimicrobial usage in humans and animals

**Figure 2:**
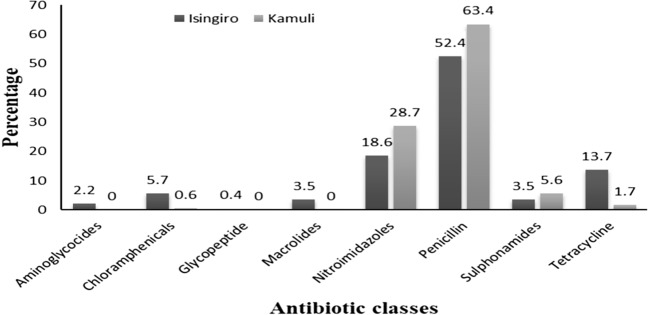
Antibiotic classes commonly used in humans in Isingiro and Kamuli districts.

The most common antibiotic classes farmers reported to use for treatment of human infections in both Isingiro and Kamuli were Penicillins and Nitroimidazoles while the least used antimicrobials were glycopeptides (Fig. 2).

**Figure 3:**
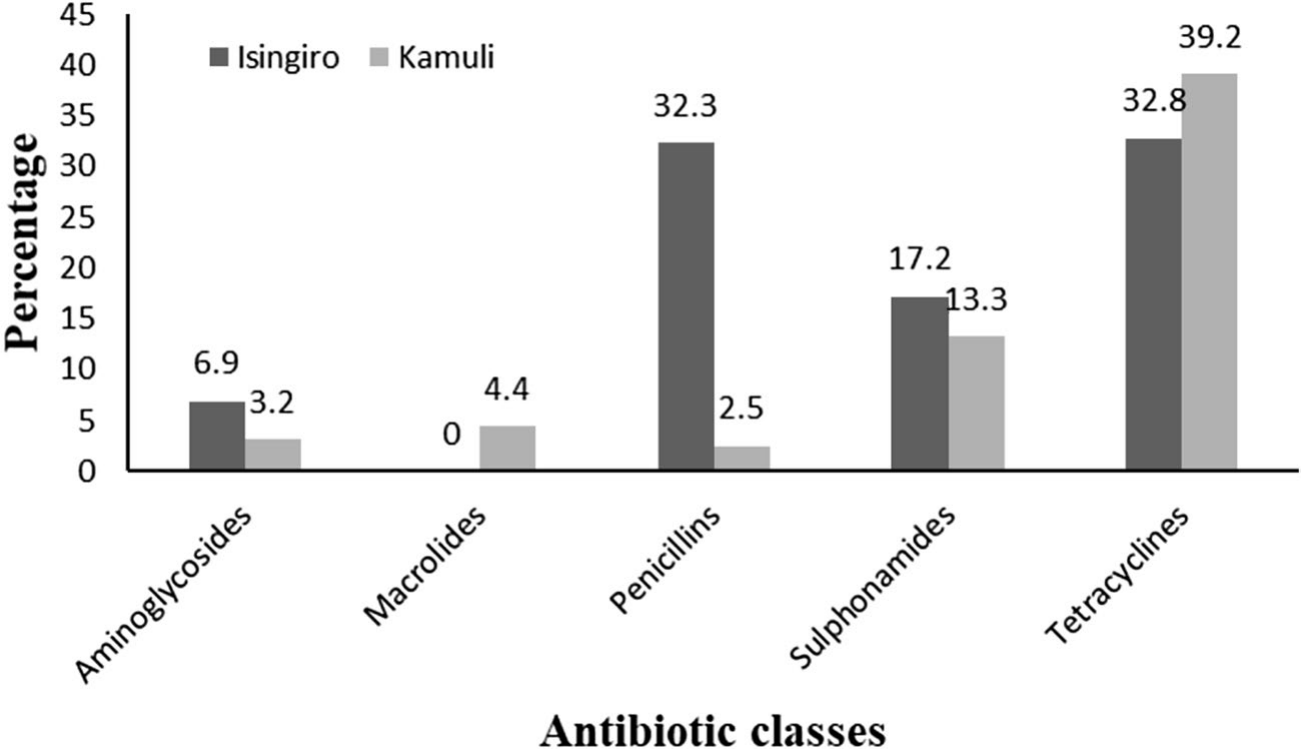
Antibiotic classes commonly used in animals in Isingiro and Kamuli districts.

While both sulphonamides and tetracyclines were noted to be popularly used in both districts for treating animal infections, penicillins were popular with Isingiro famers but not those in Kamuli district. (Fig. 3).

## Discussion

Availability of empirical data on burden of AMR and its transmission dynamics play a pivotal role in guiding policy formulation geared towards its mitigation in Uganda and globally. This study determined the prevalence of AMR in *S. aureus* among humans and animals as well as farmers’ KAPs in two different farming systems (mixed and agro-pastoral) in Uganda.

The study revealed a low prevalence of *S. aureus* (9.5%) among cattle compared to previous studies which reported prevalence of 20.3% in Uganda and 25.5% in Ethiopia^17–19^. A higher prevalence (30.6%) was observed in humans compared to previous findings in Ethiopia (28.2%), Lebanon (23.8%) and Spain (23%)^17,20,21^. The variation in the findings could probably be attributed to the different study settings, whereby this study particularly focused on apparently healthy individuals in farming communities. *Staphylococcus aureus* is considered one of the six leading opportunistic pathogens of livestock and humans responsible for AMR associated infections, hence the importance of these prevalence findings^7,22^.

This study revealed that *S. aureus* isolates from both cattle and humans exhibited antimicrobial resistance (AMR) to at least one of the assessed antibiotics, with a very high percentage showing evidence of multidrug resistance (MDR), especially to Nitroimidazoles and Penicillins. These findings are in agreement with a study which reported 100% MDR and resistance to at least two antibiotics among human isolates in a hospital survey in Ethiopia^17^. The prevalence profiles of AMR reported here are similar to those by Tibebu et al.^19^ who reported 94% prevalence to penicillin and 92% to ampicillin for cattle isolates. Likewise, a study by Rao et al.^23^ reported AMR prevalence of 81.6% to Penicillin among human isolates. The level of resistance of both cattle and human *S. aureus* isolates to Fluoroquinolones and Aminoglycosides was relatively low, a finding which was similar to that reported by Tibebu et al.^19^. The AMR prevalence findings of this study could probably be explained by the trend of routine injudicious overuse of particular antibiotics compared to others. This was noted in the current study whereby farmers reported that most common antibiotic classes they used for treatment of both cattle and human infections were Penicillins and Nitroimidazoles while the least used antimicrobials were glycopeptides and aminoglycosides.

This study revealed that several farmers in the study communities had good perceptions about AMR, with higher proportion of respondents from the mainly cattle keeping Isingiro district indicating a better understanding of the statements about AMR than those from the mixed farming setting of Kamuli district. These differences could be attributed to the value attached to cattle keeping as a source of livelihood^24–26^. A similar study in an agropastoral setting of North Western Ethiopia also reported a high (90.1%) level of knowledge of antibiotics and antibiotic resistance among animal farm owners^26^.

Most of the farmers reported undertaking good practices associated with mitigation of AMR. These included consulting qualified professionals whenever their animals were sick, routine spraying against ectoparasites, vaccination and following drug withdrawal periods by not consuming milk from lactating cows for > 7 days after treatment. Such practices were also reported to be commonly used by farmers elsewhere as measures to reduce antibiotic use, hence, contributing to mitigation of AMR^5,26–28^.

## Conclusion

This community study documents evidence ofc AMR and MDR among *S. aureus* isolated from farmers and their cattle in Isingiro and Kamuli districts. The drug classes with the highest AMR were Nitroimidazoles and Penicillins and those with the lowest levels of resistance were Fluoroquinolones and Aminoglycosides. Most farmers in this study had good knowledge of AMR, with a significantly higher proportion of respondents from Isingiro showing a better understanding of AMR than those from Kamuli. This study provides information that will be useful in drawing mitigation strategies for AMR in the study districts and Uganda as a whole.

### Supplementary Information

Supplementary Information.

## Data Availability

All relevant data used for preparation of the manuscript has been submitted as a supplementary file (File 1: Combined AMR dataset.xlsx).

## Abbreviations

AMR: Antimicrobial resistance
ESBL: Extended-spectrum beta-lactamase
MRSA: Methicillin-resistant *staphylococcus aureus*
